# Neuroinflammation and insulin resistance in major depression and bipolar disorder: Implications for clinical trials evaluating immunometabolic targeted therapies

**DOI:** 10.1016/j.bbih.2025.101166

**Published:** 2025-12-22

**Authors:** Folkert H. van Bruggen, Roger S. McIntyre

**Affiliations:** a Department of Primary and Long-Term Care, University Medical Centre Groningen, University of Groningen, P.O. Box 196, 9700 AD, Groningen, the Netherlands; b Department of Psychiatry, University of Toronto, Toronto, Ontario, Canada; c Department of Pharmacology and Toxicology, University of Toronto, Toronto, Ontario, Canada

**Keywords:** Bipolar disorder, Major depressive disorder, Neuroinflammation, Insulin resistance, Immunometabolic biomarkers

## Abstract

Bipolar disorder (BD) and major depressive disorder (MDD) are highly prevalent, disabling psychiatric illnesses marked by substantial heterogeneity and frequent metabolic and inflammatory comorbidities. Growing evidence implicates low-grade inflammation, immune dysregulation, and insulin resistance (IR) in the pathophysiology, progression, and treatment response of mood disorders. While numerous clinical trials have investigated immunometabolic targeted interventions, outcomes have been inconsistent, due to limited stratification of participants based on underlying biology. This perspective paper aims to identify practical biomarkers and biosignatures to guide patient selection and optimize immunometabolic trial design. We summarize evidence linking neuroinflammation and IR to illness burden, discuss clinical trials targeting these mechanisms, and highlight emerging markers, including extracellular vesicles, monocyte gene expression profiles, and neuron-derived vesicle signatures of IR. No single validated biomarker for identification of immunometabolic phenotype currently exists, but multimodal biosignatures combining genetic, epigenetic, proteomic, and clinical features offer a pragmatic empirical path forward. Integrating these markers with advanced analytic approaches, such as machine learning, holds promise for identifying biologically coherent subgroups most likely to benefit from targeted immunometabolic interventions, accelerating precision medicine for BD and MDD.

## 1 Introduction

Bipolar disorder (BD) is a chronic and disabling psychiatric condition affecting 1–3 % of the population, characterized by recurrent episodes of mania, depression, and mixed states, and associated with high mortality and comorbidity (McIntyre et al., 2020). Growing evidence implicates low-grade immunoinflammatory dysregulation in the onset, symptomatology, comorbidities, and treatment response of BD (Goldstein et al., 2009; Lee et al., 2021).

Alterations in immunoinflammatory markers are a consequence of multiple factors, including early-life stress and physiological vulnerability. In parallel, metabolic dysfunction—particularly insulin resistance (IR) and diabetes mellitus—has also been implicated in the pathophysiology of BD as well as depressive disorders (McIntyre, 2021; McIntyre et al., 2019). Results from both epidemiological and clinical studies have documented relatively higher rates of both inflammatory and metabolic comorbidities among individuals with the disorder (Jawad et al., 2023; Liu et al., 2022). Moreover, there is evidence to suggest that the presence of IR may be relevant to treatment outcomes in major depressive disorder (MDD) (Krupa et al., 2024).

While these observations provide a strong rationale for repurposing or developing therapies that target inflammatory and metabolic systems (Gill et al., 2021; Mansur et al., 2021), clinical trials evaluating such interventions have produced inconsistent outcomes. A key contributing factor is the biological heterogeneity of the enrolled populations and the frequent absence of stratification according to immunometabolic profiles. The inclusion of all individuals with BD or MDD, irrespective of underlying immune or metabolic alterations, likely attenuates treatment effects and reduces the interpretability and generalizability of trial findings.

The aim of this paper is to identify biomarkers that can guide patient stratification and treatment development in immunometabolic research, rather than to provide a comprehensive review of all related literature. We seek to support trialists by highlighting clinically actionable markers, specifically by (1) outlining evidence linking neuroinflammation and IR to BD, (2) summarizing findings from clinical trials targeting these mechanisms, and (3) recommending practical biomarkers and biosignatures for enriching study samples with biologically relevant subgroups.

## 2 Neuroinflammation

Neuroinflammatory processes are implicated in the pathophysiology of BD, with 20–40 % of patients showing alterations in inflammatory biomarkers. Peripheral immune activation increases blood–brain barrier permeability, allowing peripheral cells and proteins access to the central nervous system (CNS), while microglial activation and locally produced cytokines further contribute to the pathophysiology of BD (Mansur et al., 2020; Mansur et al., 2021; Siegel et al., 2021). Emerging evidence supports an immunometabolic subtype of BD, in which therapeutic development may be most effective when targeting patients with clear evidence of central immune-inflammatory activation (Lee et al., 2018). Such biomarker-guided stratification is preferable to enrolling all BD patients regardless of inflammatory status (Alageel et al., 2018; Barichello et al., 2020; Llach et al., 2025; Llach et al., 2025; Pereira et al., 2021).

In MDD, converging evidence likewise supports the presence of low-grade neuroinflammation. Large meta-analyses of peripheral blood markers have shown elevated levels of CRP, IL-6, TNF-α and other cytokines in patients with depression compared with controls, with substantial heterogeneity suggesting an “inflamed” subgroup (Osimo et al., 2020). Molecular imaging work using TSPO PET has demonstrated increased TSPO binding—interpreted as microglial activation—in anterior cingulate cortex, hippocampus, insula and prefrontal cortex in MDD, and in some studies this elevation correlates with symptom severity (Eggerstorfer et al., 2022; Setiawan et al., 2015). Complementing these findings, systematic reviews and meta-analyses of cerebrospinal fluid biomarkers report higher CSF IL-6 and other inflammatory changes in unipolar depression, alongside broader alterations in neuroendocrine and neuroplasticity-related proteins consistent with central immune activation and blood–brain barrier involvement (Enache et al., 2019; Mousten et al., 2022).

## 3 Insulin resistance and diabetes

IR and type 2 diabetes are common in BD. Treatment-naïve patients show a 2–3-fold higher risk compared to the general population (Li et al., 2024). Meta-analyses report metabolic syndrome, characterized by IR, in 33 % of BD patients vs. 11 % of controls (Tao et al., 2022) and 37 % prevalence overall, with nearly double the risk (OR 1.98, 95 % CI 1.74–2.24) compared to controls (Vancampfort et al., 2013). Beyond increased prevalence, IR in BD is linked to adverse illness course and outcomes. A systematic review and meta-analysis found that BD patients with IR or impaired glucose metabolism show worse cognitive performance (particularly verbal memory and executive function), reduced hippocampal volume, altered prefrontal neurochemistry, a more chronic or rapid cycling course, and poorer response to mood stabilizers compared to euglycemic patients (Miola et al., 2023).

In MDD, metabolic disturbances are overrepresented, with epidemiological data showing a 38–60 % increased risk of developing type 2 diabetes and a reciprocal 15 % increased risk of depression in individuals with diabetes, indicating a bidirectional relationship (Mezuk et al., 2008; Rotella and Mannucci, 2013). Independent of overt diabetes, IR is more prevalent in MDD than in non-depressed controls and has been linked to atypical symptom profiles, greater illness burden, and differential—or even lack of—response to antidepressant therapy, supporting the existence of a metabolic subtype within MDD therapy (Krupa et al., 2024; Krupa et al., 2024).

Insulin signaling within the brain is essential for maintaining cognitive function, emotional regulation, and neuroplasticity. Increasing evidence suggests that disturbances in this signaling pathway, along with impaired glucose metabolism in the brain, may contribute to the development of BD. Early studies have shown that metabolic activity in the brain varies with mood states, indicating that BD may involve disruptions in energy balance at the neuronal level. This supports the view of BD as a disorder characterized by instability in both mood and metabolic regulation (Khayachi et al., 2025).

In addition to directly targeting insulin signaling pathways, there is also a rationale for addressing related symptom domains that are structurally and functionally interconnected with the insulin receptor and its substrates. For example, GLP-1 receptor agonists are currently being explored for the treatment of bipolar disorder, MDD, schizophrenia, cognitive impairment, and substance and alcohol use disorders (McIntyre et al., 2025). Also, adjunctive liraglutide treatment in patients with MDD may improve cognitive function, in addition to clinically meaningful weight loss. These cognitive changes were partly mediated by alterations in brain structure, highlighting the connection between body weight and brain morphology and function (Mansur et al., 2017)

## 4 Immunometabolic targeted therapies in clinical trials

During the past decade, it has been amply documented that mechanistically dissimilar immunometabolic interventions hold promise to improve overall symptoms and possibly domain specific symptoms (e.g., anhedonia, general cognitive systems) in persons with mood disorder (Alageel et al., 2018; Cha et al., 2016; Lee et al., 2018; Mansur et al., 2020; Rosenblat et al., 2016; Shariq et al., 2018; Subramaniapillai et al., 2017). Additional lines of evidence in support of this thesis are provided by systematic reviews and meta-analyses of the data.

For instance, the insulin-sensitizing drug pioglitazone has been shown to improve depressive symptoms in patients with MDD (Colle et al., 2016). Similarly, statins, known for their LDL-cholesterol–lowering and anti-inflammatory properties, have demonstrated beneficial effects on depression-related outcomes when used as adjunctive therapy to SSRIs in patients with MDD (De Giorgi et al., 2021). Additionally, anti-inflammatory therapies—such as NSAIDs and cytokine inhibitors—have shown efficacy in improving depressive symptoms in patients with MDD (Köhler et al., 2014). Also, a moderate antidepressant effect was observed when anti-inflammatory agents were used as an adjunct to conventional therapy in the treatment of BD (Rosenblat et al., 2016).

While several immunometabolic therapies show promise in improving depression-related outcomes, their clinical relevance appears limited based on current systematic reviews. Moreover, other systematic reviews and meta-analyses have reported no overall significant improvement in depressive symptoms with antidiabetic agents or TNF-α inhibitors (Bavaresco et al., 2020; Nibber et al., 2022). However, individual trial analyses suggest that these null findings may largely reflect biological heterogeneity within study populations. For example, pioglitazone in BD improved IR but not psychiatric outcomes, likely due to heterogeneity in baseline inflammatory status (Aftab et al., 2019). Similarly, simvastatin failed to improve overall symptoms in early schizophrenia, yet subgroups with elevated inflammation showed benefits, and insulin receptor expression predicted cognitive outcomes (Aichholzer et al., 2022; Sommer et al., 2021; Zaki et al., 2024). In BD, infliximab did not reduce depression overall but improved outcomes in patients with childhood trauma histories (McIntyre et al., 2019). By contrast, metformin demonstrated substantial clinical benefits in treatment-resistant BD with confirmed IR, particularly among those who converted from insulin-resistant to insulin-sensitive states (Calkin et al., 2022). Together, these findings highlight that inconsistent results across trials may largely reflect biological heterogeneity and that stratification based on immunometabolic markers is essential to uncover therapeutic effects and optimize trial design.

## 5 Subphenotyping unique responses to metabolic and inflammatory treatments *neuroinflammation*

Growing evidence indicates that multiple distinct inflammatory patterns may contribute to the pathophysiology of BD and MDD, suggesting that identifying these subgroups could be essential for understanding differential treatment responses.

Monocyte inflammation-related gene expression—used as a marker of systemic inflammation—may serve as a predictor of neuroinflammatory status. This was demonstrated in a secondary analysis of a double-blind, placebo-controlled randomized controlled trial investigating simvastatin augmentation in patients with schizophrenia. While no overall symptom improvement was observed in the full study population, patients who exhibited an inflammatory profile at baseline did show clinical improvement (Aichholzer et al., 2022).

Also, a promising role appears to be reserved for extracellular vesicles (EVs). These nanoscale, membrane-bound particles are released by nearly all cell types and act as molecular biosignatures, reflecting the physiological or pathological state of their cells of origin and offering a non-invasive peripheral window into central nervous system (CNS) activity. In MDD, differentially expressed EV miRNAs have been linked to critical processes including signal transduction, neurotransmission, synaptic plasticity, and neuroinflammation. An extensive systematic review on EVs highlighted their potential both in diagnosing MDD and BD, and in identifying underlying neuroinflammatory processes. Also, there is potential in predicting response to antidepressive therapy (Llach et al., 2025).

One study highlighted the potential of EVs as peripheral biomarkers reflecting neuroinflammatory mechanisms relevant to treatment response in depression and BD. Pre-treatment levels of EV-derived microRNAs that regulate toll-like receptor 4 (TLR4) signaling—such as let-7e, miR-145, miR-146a, and miR-155—were found to discriminate between patients who would or would not remit following antidepressant therapy. These microRNAs increased with successful treatment, suggesting they may track resolution of neuroinflammation (Hung et al., 2021).

Additional EV studies revealed elevated astrocyte-derived inflammatory markers—such as glial fibrillary acidic protein (GFAP), S100β, and interleukins (IFN-γ, IL-4)—at baseline in patients with treatment-resistant depression (Xie et al., 2023; Xu et al., 2023). These levels declined following electroconvulsive therapy, supporting the hypothesis that astrocyte-driven inflammation contributes to depressive pathology.

In bipolar depression, treatment with infliximab (a tumor necrosis factor-alpha [TNF-α] antagonist) altered signaling pathways related to TNF-α and nuclear factor kappa B (NF-κB) in neuron-derived EVs. These inflammatory signatures were associated with symptom improvement, particularly in patients with a history of childhood trauma, suggesting that inflammation-related EV markers may predict and mediate treatment response in a biologically defined subgroup (Lee et al., 2021; Ran et al., 2022).

Finally, peripheral inflammatory markers may not consistently represent neuroinflammatory activity in individuals with depression. For example, while CRP levels in plasma and CSF may correlate, other markers like IL-6 and TNF-α do not. Additionally, plasma CRP is not associated with BBB integrity or depressive symptom severity, whereas CSF cytokine profiles show stronger associations with key depressive symptoms such as anhedonia and reduced motivation. Moreover, the observed negative correlation between peripheral CRP levels and imaging of brain translocator protein expression, which has been reported to be elevated in multiple brain regions in MDD further highlights the disconnect between systemic and central inflammatory activity (Llach et al., 2025).

### 5.1 Insulin resistance

Peripheral IR can be assessed using well-established clinical methods, most commonly through the Homeostatic Model Assessment of Insulin Resistance (HOMA-IR), which evaluates insulin-stimulated glucose uptake and/or glucose tolerance. However, HOMA-IR does not provide information about central nervous system (CNS) IR or CNS insulin levels. This is due to several factors: (1) insulin transport across the BBB is saturable, making CNS insulin levels non-linearly related to peripheral levels; (2) the CNS is not directly involved in the classical feedback loop regulating insulin secretion; and (3) the effects of CNS insulin on peripheral glucose and insulin regulation differ fundamentally from those of peripheral insulin (Rhea et al., 2022).

Assessing CNS IR is considerably more challenging and lacks the standardization seen in peripheral measurements. Current approaches include comparing cerebrospinal fluid (CSF) to serum insulin ratios. However, unlike HOMA-IR for peripheral IR, there is no established threshold for CSF-to-serum insulin ratios that defines CNS IR (Rhea et al., 2022).

A promising peripheral biomarker for CNS IR is the analysis of neuron-derived extracellular vesicles (NEVs) isolated from plasma, which can reveal molecular signatures of IR—though this may vary depending on the stage of the disorder. While one study found no differences in an IR–related phosphorylation ratio in drug-naïve patients with first-episode MDD (Singh et al., 2023), another reported elevated IRS-1 expression in NEVs from chronically depressed patients, most of whom were receiving psychotropic medication. These elevated levels were associated with greater severity of specific depressive symptoms (Nasca et al., 2021).

The triglyceride-to-HDL cholesterol (TG/HDL) ratio, already established as a reliable marker of peripheral IR (Baneu et al., 2024), may also serve as a predictor of central IR. In a longitudinal cohort study of individuals without baseline depression or anxiety, the TG/HDL ratio was found to predict the risk of developing MDD, although the strength of this association was moderate (hazard ratio = 1.89, 95 % CI: 1.153.11) (Watson et al., 2021). Other recent population studies demonstrated that TG/HDL-C was associated with an increased risk of depression after adjusting for all covariates (Han, 2022; Tang et al., 2025).

## 6 Discussion and conclusions

It is well-established that individuals living with BD and MDD are disproportionately affected by metabolic and inflammatory dysregulation. However, the clinical heterogeneity of these disorders, and the inconsistent results to date of trials investigating immunometabolic targeted therapies, highlight that future trials should move beyond broad inclusion strategies. The critical question is not whether inflammation and metabolism are relevant—they clearly are—but rather which biomarkers are most feasible and informative for guiding clinical trial design and identifying responsive subgroups.

Importantly, all participants must first meet established DSM-5 diagnostic criteria for BD or MDD, most often in a depressive phase. Yet the more challenging issue is whether enrichment should rely on a single marker or a broader biosignature. Reliance on isolated indicators, such as CRP elevation, is overly simplistic and unlikely to capture the biological heterogeneity of these populations. Instead, stratification will likely require integrated biosignatures that combine genetic, epigenetic, proteomic, and cellular markers as outlined in Table 1 complemented by phenotypic features such as obesity, diabetes, hypertension, or other clinical histories. It should be noted that mitochondrial dysfunction and ketone metabolism are increasingly being investigated as potential therapeutic targets in BD (Scaini et al., 2021). However, the present paper focuses on the identification of immunometabolic features that may guide the development of targeted interventions.

**Table 1:** Summary of biomarkers relevant for identifying the immunometabolic phenotype in mood disorders.

Biomarker	Clinical Relevance
Neuro inflammation	
Monocyte inflammation related gene expression	Peripheral marker of systemic inflammation; predictive of neuroinflammatory status
EV derived MiRNAs (let 7e-, MiR-145, MiR-146a, MIR-155)	Predict antidepressant response; track resolution of neuroinflammation
Astrocyte derived markers (GFAP, S100B, IFN-y, IL-4)	Elevated in treatment-resistant depression; decline with successful therapy
NF-kB signaling in EVs	Associated with symptom improvement in BD, particularly in trauma-exposed patients
CSF cytokines	Correlate with key depressive symptoms (e.g., anhedonia, low motivation)
Insulin resistance	
NEV derived IRS-1 expression	Associated with depressive symptoms severity in chronic MDD; marker of central IR
Triglyceride to HDL ratio	Peripheral proxy of IR; associated with risk of MDD and associated with CNS dysfunction

Abbreviations: BD, bipolar disorder; MDD, major depressive disorder; IR, insulin resistance; CSF, cerebrospinal fluid; EV, extracellular vesicle; NEV, neuron-derived extracellular vesicle; miR, microRNA; GFAP, glial fibrillary acidic protein; S100β, calcium-binding protein B; IFN-γ, interferon-gamma; IL, interleukin; NF-κB, nuclear factor kappa B; IRS-1, insulin receptor substrate-1; TG/HDL, triglyceride-to-high-density lipoprotein cholesterol ratio; CNS, central nervous system.

At present, no single validated biomarker exists, but assembling multimodal immunometabolic biosignatures -ideally incorporating at least one of each category as outlined in Table 1- without implying superiority of any individual biomarker, offers a pragmatic empirical path forward. The integration of such markers, interrogated with machine learning and other unbiased analytic approaches, may ultimately enable the identification of biologically coherent subgroups most likely to benefit from immunometabolic interventions (Donnelly et al., 2025; Poletti et al., 2021).

## CRediT authorship contribution statement

**Folkert H. van Bruggen:** Writing – review & editing, Writing – original draft, Conceptualization. **Roger S. McIntyre:** Writing – review & editing, Supervision, Conceptualization.

## Funding

This article was written without external financial support.

## Declaration of competing interest

Folkert van Bruggen: none.

Roger McIntyre has received research grant support from CIHR/GACD/National Natural Science Foundation of China (NSFC) and the Milken Institute; speaker/consultation fees from Lundbeck, Janssen, Alkermes, Neumora Therapeutics, Boehringer Ingelheim, Sage, Biogen, Mitsubishi Tanabe, Purdue, Pfizer, Otsuka, Takeda, Neurocrine, Neurawell, Sunovion, Bausch Health, Axsome, Novo Nordisk, Kris, Sanofi, Eisai, Intra-Cellular, NewBridge Pharmaceuticals, Viatris, Abbvie and Atai Life Sciences.

## Data Availability

No data was used for the research described in the article.
